# Comparing different maize supplementation strategies to improve resilience and resistance against gastrointestinal nematode infections in browsing goats[Fn FN1]

**DOI:** 10.1051/parasite/2015019

**Published:** 2015-06-12

**Authors:** Leslie Gárate-Gallardo, Juan Felipe de Jesús Torres-Acosta, Armando Jacinto Aguilar-Caballero, Carlos Alfredo Sandoval-Castro, Ramón Cámara-Sarmiento, Hilda Lorena Canul-Ku

**Affiliations:** 1 FMVZ, Universidad Autónoma de Yucatán, Km 15.5 Carretera Mérida-Xmatkuil Mérida Yucatán 97315 Mexico; 2 FMVZ, Universidad Michoacana de san Nicolás de Hidalgo, Unidad Acueducto, Av. Acueducto Esq. Tzintzuntzan Col. Matamoros Morelia Michoacán 58130 Mexico

**Keywords:** Browsing goats, Energy supplementation, Resilience, Resistance, Gastrointestinal nematodes, Alternative control

## Abstract

The effect of maize grain supplementation on the resilience and resistance of browsing Criollo goat kids against gastrointestinal nematodes was evaluated. Five-month-old kids (*n* = 42), raised worm-free, were allocated to five groups: infected + not supplemented (I-NS; *n* = 10), infected + maize supplement at 108 g/d (I-S108; *n* = 8), maize supplement at 1% of body weight (BW) (I-S1%; *n* = 8), maize supplement at 1.5% BW (I-S1.5%; *n* = 8), or infected + supplemented (maize supplement 1.5% BW) + moxidectin (0.2 mg/kg BW subcutaneously every 28 d) (T-S1.5%; *n* = 8). Kids browsed daily (7 h) in a tropical forest for 112 days during the rainy season. Kids were weighed weekly to adjust supplementary feeding. Hematocrit (Ht), hemoglobin (Hb), and eggs per gram of feces were determined fortnightly. On day 112, five goat kids were slaughtered per group to determine worm burdens. Kids of the I-S1.5% group showed similar body-weight change, Ht and Hb, compared to kids without gastrointestinal nematodes (T-S1.5%), as well as lower eggs per gram of feces and *Trichostrongylus colubriformis* worm burden compared to the I-NS group (*P* > 0.05). Thus, among the supplement levels tested, increasing maize supplementation at 1.5% BW of kids was the best strategy to improve their resilience and resistance against natural gastrointestinal nematode infections under the conditions of forage from the tropical forest.

## Abstract (fr)

L'effet de la supplémentation en grains de maïs sur la résilience et la résistance de chevreaux de race Criollo au pâturage contre les nématodes gastro-intestinaux a été évalué. Des chevreaux âgés de cinq mois (*n* = 42), élevés sans vers parasites, ont été partagés en cinq groupes : infesté + non supplémenté (I-NS ; *n* = 10), infesté + supplément de maïs à 108 g/j (I-S108 ; *n* = 8), supplément de maïs à 1 % du poids vif (PV) (I-S1 % ; *n* = 8), supplément de maïs à 1.5 % PV (I-S1.5 ; *n* = 8) ou infesté + (supplément de maïs 1.5 % PV) + moxidectine (0.2 mg/kg PV sous-cutané tous les 28 jours) (T-S1.5 % ; *n* = 8). Les chevreaux pâturaient chaque jour (7 h) dans une forêt tropicale pendant 112 jours en saison des pluies. Les chevreaux ont été pesés chaque semaine pour ajuster l'alimentation complémentaire. L’hématocrite, l'hémoglobine et les œufs par gramme de fèces ont été déterminés par quinzaine. Au jour 112, cinq chevreaux par groupe ont été abattus afin de déterminer la charge parasitaire. Les chevreaux du groupe I-S1.5 % ont montré des changements de poids vif, hématocrite et hémoglobine similaires aux chevreaux sans nématodes gastro-intestinaux (T-S1.5 %), ainsi que des valeurs d’œufs par gramme de selles et une charge en *Trichostrongylus colubriformis* inférieurs à celles du groupe I-NS (*P* > 0.05). Ainsi, parmi les niveaux de suppléments testés, la supplémentation en maïs à 1.5 % du poids vif des chevreaux était la meilleure stratégie pour améliorer leur résilience et la résistance contre les infections naturelles par nématodes gastro-intestinaux dans les conditions de pâture de la forêt tropicale.

## 1. Introduction

Gastrointestinal nematode (GIN) infections significantly reduce growth rates of goat kids grazing or browsing in the tropical deciduous forest of Mexico [33, 34]. Although the control of GIN infection in goat production systems in Mexico relies exclusively on the frequent use of anthelmintic (AH) drugs, the sustainable control of GIN infection should reduce the use of AH and encourage the application of alternative control measures such as supplementary feeding [32, 35]. Controlled infection studies have shown that supplementary feeding improves resilience against GIN in growing kids [7, 8]. Field studies performed under browsing conditions in Yucatán, México, during the rainy and dry seasons showed that a fixed amount of supplement (100 g DM/d; 74% sorghum grain, 26% soybean meal) was an economically feasible practice to improve resilience against GIN [25, 33, 34]. However, further improvements in the effect of supplementary feeding on resilience or resistance against GIN could be obtained by targeting a supplementation strategy according to the type of vegetation available in the heterogeneous vegetation [21, 23]. Recent studies [17] showed that a large proportion of plants of the tropical deciduous forest (TDF) consist of browsing legumes with high protein and low energy content. The latter results in excess of rumen degradable nitrogen (RDN). For this reason, supplementing with a source of rumen fermentable energy (RFE), such as maize grain, could be used to take advantage of the abundant RDN that goats ingest from the legume trees present in the grazing area. Maize supplementation could have at least four positive outcomes for the goats: (a) the supplemented RFE helps in the use of RDN ingested in the browse to increase microbial protein reaching the abomasum, (b) a better RFE:RDN ratio may increase rumen production of volatile fatty acids, (c) reducing the excess RDN helps to reduce the energy cost of N excretion from the goat’s body, and (d) a certain small portion of maize starch could bypass rumen fermentation and reach post-ruminal digestion. The net outcome of maize supplementation would be to increase the quantity of nutrients for the animals. Part of these nutrients can be used to improve resilience against GIN as suggested by other studies in browsing kids [18, 24] and hair sheep lambs [30]. Meanwhile, a reduction in fecal egg counts or GIN burden (resistance) has been less evident [18, 24]. Another way to improve the effect of supplementary feeding on resilience against GIN could be to adjust the amount of supplement offered to growing animals. Previous field trials showed that the positive effect on the resilience against GIN in kids was reduced as the experiments progressed into the rainy season. These studies were carried out using fixed amounts of supplements per animal (85–100 g DM/d during the whole trial). As a result, there was a large amount of supplementary nutrients at the beginning of the growing period (as a proportion of the animal’s body weight [BW]) and a smaller amount (as a proportion) as the animals grew. A higher amount of nutrients is required toward the end of the growing period since: (i) animals become heavier and proportionally require more nutrients; (ii) more GIN infectivity is expected as the rainy season progresses, thus, a higher parasitic challenge results in an increment of the metabolic/nutritional cost; and (iii) animals could mount an immune response against GIN which also requires additional nutrients. We hypothesize that adjusting the amount of the energy supplement (as a proportion of animal BW; i.e., 1 or 1.5% of BW) could solve the problem of nutrient supply explained above. Therefore, the objective of the present experiment was to compare the effect on resilience and resistance against GIN either from a fixed supplementation level (100 g DM/d) or from supplementation that increases as a proportion of the animal’s live weight (1 or 1.5% BW).

## 2. Materials and methods

### 2.1. Study area

A field study was performed over 112 days of the rainy season (from August to December) at the small ruminant farm of the FMVZ-Universidad Autónoma de Yucatán, México (20°52′07″ N and 89°37′24″ W). The climate of the study area is AW_0_ (tropical warm sub-humid with summer rainfall) with a mean average temperature of 26 °C, humidity of 72%, and annual rainfall of 1100 mm. A nearby, semi-deciduous tropical forest, with abundant native leguminous shrubs and trees was used for grazing the experimental animals [15]. A list of the native vegetation in the study area can be obtained from Ríos and Riley [31] and González-Pech et al. [17].

### 2.2. Animals and experimental treatments

A total of 42 five-month-old Criollo kids (castrated males and females) were used (average BW of 15 ± 1.92 kg). Kids were raised and maintained free of GIN infection before the trial started as described by Torres-Acosta et al. [33]. At the beginning of the trial, animals were distributed into five groups balanced for BW and sex: The I-NS group with 10 naturally infected kids, non-supplemented; the I-S108 group with 8 naturally infected kids, supplemented daily with ground maize grain at 108 g fresh basis (FB); the I-S1% group with 8 naturally infected kids, supplemented daily with ground maize grain at 1% BW FB; the I-S1.5% group with 8 naturally infected kids, supplemented daily with ground maize grain at 1.5% BW FB, and the T-S1.5% group with 8 kids treated with moxidectin (Cydectin^®^) every 28 d and supplemented daily with ground maize grain at 1.5% BW FB.

Nutritional characteristics of browsing forage and the supplement (maize grain) are presented in Table 1. The nutritional components of the browse vegetation were calculated from a database containing the chemical composition and degradability of leafs from different browse plants consumed by goats in the tropical deciduous forest (*Leucaena leucocephala*, *Acacia pennatula*, *Havardia albicans*, and *Piscidia piscipula*). The estimated metabolizable energy (ME) and metabolizable protein (MP) requirements for a 15 kg kid and the proportion of nutrient supply from the supplement in the different experimental groups [1] are presented in Table 2.

**Table 1.** Chemical composition (g/kg DM, except where stated) and feed degradation parameter considered to estimate nutrient supply of browsing vegetation and maize grain for Criollo kids naturally infected with gastrointestinal nematodes (GIN) during the rainy season.

Nutritional components	Browse vegetation g/kg DM	Maize g/kg DM
Crude protein	180	80
Ether extract	40	30
Metabolizable energy (MJ/kg DM)	9	13
Acid detergent insoluble nitrogen	1	1
Constants*		
“A” (Degradation (loss) at time 0)	0.39	0.30
“a”	0.29	0.35
“b”	0.55	0.60
“c”	0.100	0.065

* According to the model *P* = *a* + *b* (1 − exp^(−ct)^). AFRC [1].

**Table 2.** Maintenance requirements of metabolizable energy (ME; MJ/d) and metabolizable protein (MP; g/d) for 15 kg BW kids (50 g/d of BW gain, walking 10 km/d), and nutrient supply from supplement (ground maize grain fresh basis) in the respective experimental groups (including the % of the requirement covered with the supplement) (as per AFRC, 1993).

	ME	% of requirement	MP*	% of requirement
Nutrient requirement for a 15 kg BW kid	5.96	100%	31.44	100%
Nutrient supply from maize supplement				
Group I-S108 (108 g maize)	1.3	21.8	5.38	17.1
Group I-S1% (150 g maize)**	1.76	29.5	7.27	23.1
Group I-S1.5% (225 g maize)**	2.64	44.3	10.90	34.7
Group T-S1.5% (225 g maize)**	2.64	44.3	10.90	34.7

* For the supplement, the potential MP supply was estimated on the basis of the rumen fermentable energy and the bypass digestible protein.

** Quantity of maize used to calculate the nutrient supply per group.

### 2.3. Animal management

*Supplementation.* With the exception of kids in the I-S108 group, which were individually fed 108 g ground maize grain during the whole experiment, the amounts of supplement were adjusted at weekly intervals according to the experimental group. Intake of maize supplement was verified every day and refusals were measured before animals were released to the field for browsing.

*Browsing management.* Before the beginning of the experiment, kids were taken to the fields of tropical semi-deciduous forest for adaptation (1 week). After that period, animals were grazed daily from 7:00 a.m. to 2:00 p.m. (7 h/d). Grazing was performed in naturally infected fields of the tropical semi-deciduous forest. Experimental animals grazed together with a herd of 200 adult animals (sheep and goats).

*Anthelmintic treatment.* Animals in the T-S1.5% group were treated with 1% moxidectin liquid formulation (Cydectin^®^ NF for cattle, Fort Dodge) administered subcutaneously at a dose of 0.2 mg/kg BW. First treatment was applied on day 0 and subsequent doses were given every 28 days.

*Fecal cultures and identification of GIN infective larvae* (*L*_3_)*.* Bulk fecal cultures were performed for each experimental group every 28 days. Cultures were kept at room temperature and were harvested seven days after using the Corticelli and Lai [12] technique. The genera of the GIN L_3_ larvae harvested from the cultures were identified using adequate keys of identification [9]. The purpose of this procedure was to monitor, indirectly, the nematode genus present within the experimental animals at different time points in the trial [25].

### 2.4. Effect of supplementation level on host resilience

*Bodyweight change.* Animals were weighed with an electronic weighing scale on day 0 and at weekly intervals for the duration of the experiment. Weighing took place in the mornings before the kids went out to browse (after 16 h of fasting). The cumulative BW change (CBWC) was obtained as the difference between the initial and the final BW.

*Hemoglobin* (*Hb*) *and hematocrit* (*Ht*)*.* A 3 mL blood sample was taken from the jugular vein (18 × 1.5 in needles) of each kid on day 0 and every 14 d thereafter. Concentration of Hb was determined by the cyanmethemoglobin method, while Ht was measured using the capillary microhematocrit method [6].

### 2.5. Effect of supplementation level on worm populations within hosts

*Fecal egg count* (*FEC*)*.* Individual fecal samples (*c.* 5 g) were obtained from the rectum of kids using plastic bags. Eggs per gram (EPG) was determined using a modified McMaster technique with a sensitivity of 50 eggs/g of feces.

*Post-mortem* parasitological measurements*.* Five kids were randomly selected from each experimental group to recover their GIN population. The humane slaughter of kids from different experimental groups was performed according to the corresponding Official Mexican Standard NOM-033-ZOO 1995. The respective abomasum, small and large intestines were ligated independently and were removed from every animal. Organs were processed separately immediately after slaughter. The quantity of adult GIN in 10% aliquots from the contents and washings obtained from the different sections of the gastrointestinal tract (abomasum, small and large intestine) was determined as described by MAFF [27]. Worm identification was made using all the specimens found in the aliquots obtained from the abomasum, small and large intestine, respectively, using the keys of identification by Barth and Visser [5]. The female worms of the Strongylida order (*Haemonchus contortus*, *Trichostrongylus colubriformis* and *Oesophagostomum columbianum*) were measured longitudinally to determine their length and the amount of eggs *in utero* was estimated as per Martínez-Ortiz-de-Montellano et al. [25].

### 2.6. Statistical analysis

Data on BW, Ht, Hb, and EPG were analyzed using a repeated measures analysis with the GLM procedure of Minitab 15. The Tukey post hoc test was used when the null hypothesis was rejected. Before the analysis, the EPG data was log(10) transformed (EPG + 1). The EPG data analysis started from day 28 of the trial, when GIN infection was patent in all the groups (group T-S1.5% was not included as it remained at zero during the trial). The mean total CBWCs of each group were compared with the ANOVA test and with Tukey post hoc test. Respective regression analyses were performed for cumulative bodyweight gain (CBWG), daily bodyweight gain (DBWG), Ht, Hb, and the log-transformed EPG data against the individual consumption of maize supplement.

The *post-mortem* variables (total number of adult worms and female worms, female length, and number of eggs *in utero*) were checked for normality by means of respective Kolmogorov-Smirnov tests for each worm species (*H. contortus*, *T. colubriformis*, and *O. columbianum*). The *post-mortem* variables were not normally distributed, even after transformation. Thus, experimental groups were compared using the Kruskal-Wallis test (a respective test for each worm species). When a Kruskal-Wallis test was significant, pairwise comparisons were made using the Wilcoxon rank sum tests to determine which pairs of groups differed [28].

### 2.7. Economic analysis

The costs of the interventions (AH treatment and/or supplementation) were recorded and used in a partial budget analysis [19] to determine the economic viability of AH treatment and/or maize supplementation. Price of supplementary feeding with maize grain was estimated at $ 0.26 US/kg, the cost of the moxidectin treatment was $ 0.55 US/mL, and the cost of a kg of kid was $ 2.3 US/kg.

## 3. Results

Four animals from the I-NS group were withdrawn from the study before the end of the trial as they required salvage treatment. Another two animals from the I-S1.5% group were withdrawn from the study due to causes different from the study protocol.

### 3.1. Effect of supplementation level on host resilience

**Figure 1.**
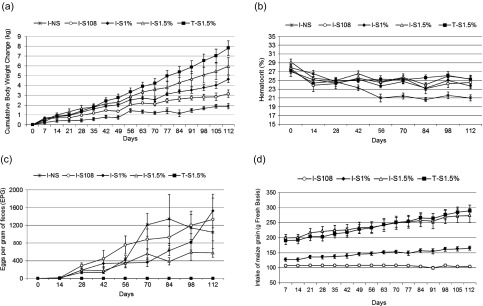
Effect of different levels of maize supplementation on the cumulative bodyweight change (kg) (a), hematocrit (%) (b), nematode eggs per gram of feces (c), and maize intake (g fresh basis) (d) in browsing Criollo kids naturally infected with gastrointestinal nematodes during the rainy season. I-S108 = infected and supplemented with 108 g maize grain. I-S1% = infected and supplemented with maize grain at 1% LW. I-S1.5% = infected and supplemented with maize grain at 1.5% LW. NI-S1.5% = no infected (moxidectin treatment) and supplemented with maize grain at 1.5% LW. I-NS = infected non supplemented

*Body-weight change and maize consumption.* The mean CBWC of different groups throughout the trial is shown in Figure 1a. The mean CBWC (kg), the daily BWC (g) of each experimental group and the respective total maize grain intake (kg) are shown in Table 3. The regression equation for CBWG (kg) was 1.2 (±0.44) + 0.02 (±0.002) g maize consumption (*R* ^2^ = 71.8%, *P* < 0.0001). The equation for DBWG (g) was 11.0 (±3.96) + 0.21 (±0.023) g maize consumption (*R* ^2^ = 71.8%; *P* < 0.0001). Supplementing at 1.5% BW either without GIN infection (group T-S1.5%) or infected (I-S1.5%) resulted in higher CBWC and daily BW gain (*P* < 0.05) than the group kept without supplement (I-NS) or the group receiving a fixed amount of supplement (I-S108 groups). The last two groups were similar in terms of growth (*P* > 0.05). Meanwhile, supplementing at 1% BW (I-S1%) also improved growth compared to the I-NS group (Table 3). Thus, supplemented groups had a BWC that was proportional to the amount of maize supplement received. The animals in the I-NS groups consistently had the lowest BWC, even losing weight at some weeks (Fig. 1a). Except for the I-S108 group that received a fixed amount of supplement, the intake of supplement increased as a proportion of weight (as planned) (Fig. 1d). The total maize intake of T-S1.5% kids was higher than those of the I-S1% and I-S108 kids (*P* < 0.05; Table 3).

**Table 3.** Mean (±SEM) of the cumulative bodyweight change (kg), daily bodyweight change (g), total maize intake (kg), hematocrit (%), hemoglobin (g/dl) and eggs per gram of feces (EPG) of browsing kids supplemented with different levels of maize grain during the rainy season.

Groups	*n*	Cumulative BWC kg (±SEM)	Daily BWC g (±SEM)	Total maize intake kg (±SEM)	Ht % (±SEM)	Hb g/dl (±SEM)	EPG (±SEM)
T-S1.5%	8	7.81 ± 0.24d	69.75 ± 6.63d	26.7 ± 1.17b	25.43 ± 0.54b	7.74 ± 0.16b	0
I-S1.5%	6	6.46 ± 0.74cd	57.72 ± 7.45cd	26.8 ± 1.43b	25.75 ± 0.63b	8.06 ± 0.19b	579 ± 82a
I-S1%	8	4.63 ± 0.83bc	41.29 ± 3.10bc	16.4 ± 1.34a	24.69 ± 0.54ab	7.51 ± 0.16b	1525 ± 381b
I-S108	8	3.16 ± 0.35ab	28.18 ± 3.45ab	11.9 ± 1.24a	24.79 ± 0.54ab	7.74 ± 0.16b	1331 ± 485b
I-NS	6	1.88 ± 0.39a	16.74 ± 2.10a	–	23.03 ± 0.63a	6.92 ± 0.194a	1042 ± 426b

Means with different letters in the same column differ at *P* < 0.05. SEM = standard error of the mean. T-S1.5% = moxidectin-treated and supplemented with maize grain at 1.5% BW. I-S1.5% = naturally infected and supplemented with maize grain at 1.5% BW. I-S1% = naturally infected and supplemented with maize grain at 1% BW. I-S108 = naturally infected and supplemented with 108 g maize grain. I-NS = naturally infected, not supplemented.

*Hematocrit and hemoglobin.* The mean Ht values of the groups supplemented at 1.5% BW (groups T-S1.5% and I-S1.5%) were significantly different from the values of the I-NS group. However, the other levels of supplementation (I-S1% and I-S108) were not significantly different to that of the I-NS group. The mean Ht of the I-NS group declined from the beginning of the experiment, stabilizing at their lowest values from day 56 onwards. On the other hand, the supplemented animals maintained their Ht values above 22% (Fig. 1b). The mean Hb values of the supplemented groups were higher than those of the I-NS group (Table 3). The regression equation for Ht (%) was 23.7 (±0.45) + 0.007 (±0.003) g maize consumption (*R* ^2^ = 17.7%; *P* < 0.007) and for Hb (g/dl) was 7.1 (±0.14) + 0.004 (±0.001) g maize consumption (*R* ^2^ = 33.0%; *P* < 0.0001).

### 3.2. Effect of supplementation level on worm populations within hosts

*Eggs per gram of feces and infective larvae.* The supplemented group I-S1.5% had the lowest fecal excretion of EPG (*P* < 0.05). Meanwhile, the other supplemented groups (I-S108 and I-S1%) did not differ in their EPG with the I-NS group (*P* > 0. 05; Fig. 1c; Table 3). The regression equation for EPG (Log EPG + 1) was 3.18 (±0.26) − 0.007 (±0.002) g maize consumption (*R* ^2^ = 35.5%; *P* < 0.0001).

Fecal cultures prepared at the end of August (first month of the trial) did not produce L3 larvae because the GIN infection was not patent. The L3 larvae obtained from the fecal cultures of the different groups at the end of September were predominantly *Haemonchus* sp. (about 75%) with small proportions of *Trichostrongylus* sp. (about 12%) and *Oesophagostomum* sp. larvae (about 17%). At the end of October, the *Haemonchus* sp. larvae were close to 45%, while *Trichostrongylus* sp. larvae were around 25% and *Oesophagostomum* sp. larvae around 30%. At the end of November, *Haemonchus* sp. were again the predominant L3 larvae (about 65%) with a low proportion of *Trichostrongylus* sp. (about 15%) and *Oesophagostomum* sp. (about 20%). At the end of the study (December), the *Haemonchus* sp. larvae were less abundant (below 10%), while the *Trichostrongylus* larvae reached above 25% and the *Oesophagostomum* sp. larvae were close to 65%.

*Adult worm burdens.* Total adult and female *H. contortus* worm burdens were not different between groups (*P* > 0.05; Table 4). However, the supplemented group (I-S1.5%) had a lower total burden of *T. colubriformis* compared with I-NS and I-S1% (*P* < 0.05). Meanwhile, female burdens of all the supplemented groups were lower than the I-NS group (*P* < 0.05; Table 4). The *O. columbianum* total and female burdens were not different in the various groups (*P* > 0.05; Table 4). Although there was no consistent statistical difference between the worm burdens of the I.S1.5% and the I-NS groups, the former group showed a reduction of 63.9% for *H. contortus*, 70.1% for *T colubriformis*, and 50% for *O. columbianum* compared to the I-NS group.

**Table 4.** Median (minimum – maximum) number of adult parasites (total and females) recovered in the different sections of the gastrointestinal tract of browsing kids supplemented with different levels of maize grain during the rainy season and the respective median length of adult female worms and median number of eggs *in utero* (minimum – maximum).

Groups	Total	Females	Length (mm)	Fecundity
*Haemonchus contortus*				
I-NS (*n* = 5)	720 (60–2110)	400 (30–1050)	19.0 (15–25)a	140 (10–570)a
I-S108 (*n* = 5)	460 (210–930)	290 (80–420)	19.0 (14–24)a	130 (10–880)a
I-S1% (*n* = 5)	270 (170–660)	160 (60–260)	18.0 (12–23)a	120 (10–470)a
I-S1.5% (*n* = 5)	260 (70–550)	130 (40–340)	20.5 (14–24)b	260 (10–710)b
*Trichostrongylus colubriformis*				
I-NS (*n* = 5)	1540 (1080–3160)a	880 (780–1700)a	7.0 (6–9)a	17 (3–34)a
I-S108 (*n* = 5)	680 (640–2120)ab	480 (160–920)a	7.5 (6–9)a	19 (3–34)ab
I-S1% (*n* = 5)	1280 (440–2040)a	680 (320–1120)a	7.5 (6–9)a	20 (5–31)b
I-S1.5% (*n* = 5)	460 (80–760)b	260 (80–360)b	8.0 (6–9)b	21 (6–37)b
*Oesophagostomum columbianum*				
I-NS (*n* = 5)	160 (30–330)	60 (10–180)	18 (15–21)a	165 (30–540)a
I-S108 (*n* = 5)	150 (40–220)	60 (20–120)	18 (16–21)ab	175 (10–920)a
I-S1% (*n* = 5)	140 (0–160)	60 (0–80)	19 (14–20)b	360 (10–770)b
I-S1.5% (*n* = 5)	80 (10–100)	25 (0–80)	19 (17–22)b	235 (140–620)a

Medians with different letters in the same column differ at *P* < 0.05. I-NS = infected, not supplemented. I-S108 = infected and supplemented with 108 g maize grain. I-S1% = infected and supplemented with maize grain at 1% BW. I-S1.5% = infected and supplemented with maize grain at 1.5% BW.

*Length and eggs* in utero *of female worms.* The I-S1.5% group had larger worms for all worm species compared to the I-NS group (*P* < 0.05). Higher numbers of eggs *in utero* were also evident for the I-S1.5% compared to the I-NS group, with the exception of *O. columbianum*.

Meanwhile, the *H. contortus* female worms recovered from the I-S1% group were similar to those of the I-NS group. The *T. colubriformis* in the I-S1% group had more eggs *in utero* than I-NS. The *O. columbianum* of this group were larger and contained more eggs *in utero* than those in the I-NS group (*P* < 0.05).

Finally, the length and number of eggs *in utero* of the three worm species recovered from the I-S108 group were similar to those obtained in the I-S1% group (Table 4).

### 3.3. Economic analysis

The mean net profit obtained per kid in the I-NS group was $ 2.6 US. The supplemented kids obtained an additional income that was sufficient to cover the cost of the supplement and moxidectin treatment (in the T-S1.5% group). The net additional income from maize supplementation was positively associated with the supplementation level: $ 1.7 US, $ 3.9 US, $ 5.8 US, and $ 7.9 per kid in groups I-S108, I-S1%, I-S1.5%, and T-S1.5%, respectively.

## 4. Discussion

### 4.1. Effect of supplementation level on host resilience

Some decades ago, Poppi et al. [29] stated that “the crucial question is whether nutrient manipulation can increase growth of parasitized animals by maintaining the supply of absorbed nutrients to productive tissues (muscle and bone) as well as providing nutrients to allow for repair of damaged tissues (gut wall), and the immunological response of the host”. This kind of nutrient manipulation, leading to improved resilience and/or resistance against GIN, is also known as an indirect effect of nutrition. The indirect effect is very much related to the supply of more nutrients to the host (supplementary feeding) helping to satisfy the increased requirements of nutrients resulting from GIN infection [21]. At present, evidence suggests that supplementary feeding can be a valuable strategy to control GIN infections by enhancing resilience of tropical goat kids [25, 33] and lambs [30]. The present study showed that the control of GIN through supplementary feeding could be made more effective by adjusting the supplementation strategy to the features of the forage available in the grazing area [23, 35]. Hence, in swards where tropical grasses are the main forage available, supplementing with protein sources has proven to be a valuable strategy [13]. Meanwhile, when animals graze or browse protein-rich vegetation, supplementation with a source of RFE (such as maize grain) can be a good strategy to improve resilience against GIN, as shown in earlier field trials with kids [18] and lambs [30]. Maize grain can help to take advantage of the abundant RDN ingested by goats from the legume trees in the tropical forest. Such high RDN could be balanced with the RFE from maize supplement to achieve at least four positive outcomes for the animals: (a) increase rumen microbial protein production reaching the abomasum, (b) increase rumen production of volatile fatty acids, (c) reduce energy cost of N excretion from the goat’s body, and (d) a certain small portion of maize starch could bypass rumen fermentation and reach post-ruminal digestion. The net outcome would be to increase the quantity of nutrients for the animals. In this study, it was evident that infected animals use part of these nutrients to improve their resilience against GIN. This is consistent with the definition of resilience [21]. Improvement of resilience was more evident when the supplement was increased up to 1.5% BW compared to a fixed amount per day (108 g/day). Thus, this study also showed that it is important to adjust the supplementation strategy in terms of quantity. Previous field trials found that the beneficial effects of supplementing with a fixed amount of feed were reduced over time, as the animals progressed in the rainy season [18, 24, 33, 34]. Similarly to previous field trials, in the present study the fixed amount of supplement was proportionately smaller (% BW) with time, which means that fewer nutrients were available to sustain improved resilience. As a result, the I-S108 group increased their CBWC 68% compared to the I-NS group. Meanwhile, the I-S1% and I-S1.5% groups showed larger increments in their CBWC compared to the I-S group (146% and 243% respectively). The latter also suggested that RFE was the limiting nutrient for BW gain. Thus, in relation to the fixed amount of maize in the I-S108 group, maize intake increased by 38%, 125%, and 124% in I-S1%, I-S1.5%, and T-S1.5%, respectively. These effects can easily be observed in Figures 1a and 1d. Matching rumen energy and protein requirements allows an increment in nitrogen retention and metabolic protein supply [14, 20]. The increased amount of energy given as supplement allowed the kids to use the protein-rich legume fodder present in the browsing area in a more efficient manner and hence better express resilience against GIN.

The present study also provided evidence of the metabolic cost of natural GIN infection. The use of a suppressive anthelmintic treatment regimen in two equally supplemented groups (I-S1.5% vs. NI-S1.5%) showed a tendency to increase the CBWC in the non-infected group (20% increment; *P* > 0.05). This difference could account for the consequences resulting from the GIN challenge (i.e*.* tissue repair, blood and plasma synthesis, immune cell production, etc.) [10, 11, 13]. Under similar conditions of mixed natural GIN infection and supplementation levels, Retama-Flores et al. [30] found an average metabolic cost of 43.5 g BWC/d in Pelibuey lambs browsing in the semi-deciduous tropical forest. This value is similar to the difference found in the present experiment (41 g BW/d) between groups I-NS and IS1.5%. The metabolic cost of the mixed GIN infection seems to be equally due to *T. colubriformis* and *H. contortus* infections (Table 4). Although the numbers of *O. columbianum* were not as high as the other two species of GIN, its nutritional/metabolic penalty should not be minimized.

The improvement of resilience was evident based on the analysis of Ht and Hb parameters, even when four kids with the lowest blood parameters were withdrawn from the I-NS group during the trial and their low blood parameters were not included in the statistical comparisons. Supplementation provided kids with additional nutrients that helped them to maintain their blood parameters within normal values for the entire rainy season, in spite of the mixed GIN infections. Similar results have been reported in previous wet season field studies with browsing kids [24, 33] and hair sheep lambs [30].

It is important to highlight that the I-S1.5% group showed better growth, Ht and Hb than the I-NS group as a result of the improved nutritional level, but also as the result of a lower GIN infection level. Thus, improved resilience cannot be claimed as the sole reason for the improved productivity of the I-S1.5% group compared to the I-NS group. The I-S1.5% group seemingly showed the effect of improved resistance against GIN as will be discussed below.

### 4.2. Effect of supplementation level on worm populations within hosts

Supplementing infected kids at 1.5% BW (I-S1.5%) resulted in a 55% reduction in EPG compared with I-NS kids (*P* < 0.05). This reduction in EPG is similar to the 69% reduction reported for maize supplemented lambs by Retama-Flores et al. [30]. This reduction effect was not significant with the other supplementation levels (*P* > 0.05). Two possible explanations can be offered for these results on EPG. Firstly, only the kids supplemented at 1.5% BW seemed to have the nutrient supply necessary to mount an adequate immune response to affect their GIN population (resistance). As explained above, the immune response could benefit from the extra nutrients from supplementation. Secondly, the supplementation level could have reduced the ingestion of fodder material in the field. Hence, the number of infective larvae consumed by these supplemented animals could have been reduced (compared to the I-NS group and possibly the I-S108 group as well). The reduction in fodder ingestion due to the consumption of a supplement has been reported in Pelibuey lambs through the observation of their feeding behavior in the field [30].

The adult GIN worm burdens (females and total) tended to be reduced as the supplementation level increased, especially with *H. contortus* and *O. columbianum* (Table 5). However, due to the small number of animals used in the comparison, such differences were not significant. The only exception was found in the I-S1.5% group for *T. colubriformis* that showed a significant reduction of total and female worm burdens compared to the I-NS group. A reduced GIN burden and the resulting lower EPG in the I-S1.5% group suggested an improved immune response, which can only be expressed with an adequate nutritional condition [2].

A trend was observed to obtain the largest and most fecund GIN in the I-S1.5% group. These effects might be due to the smaller worm populations found in this group, which allowed them more space (and possibly access to nutrients) to achieve a larger size and fecundity [3, 4]. On the other hand, a smaller length or fecundity in the I-NS group could have resulted from eating more fodder from the tropical forest compared to the supplemented kids. Several fodder materials of the tropical forest contain high concentrations of secondary compounds (SCs), which can induce a reduction in the length and fecundity of the female worms, as shown in artificial infection pen trials [16, 26]. The SCs may affect worm nutrition or egg production/excretion which can alter, either indirectly or directly, nematode reproductive function [22].

### 4.3. Economic analysis

Although the kids in the T-S1.5% (without a patent GIN infection) achieved the best performance and higher economic viability, the suppressive AH scheme (moxidectin every 28 d) is not a practical option. This type of treatment regimen can generate anthelmintic resistance in the short term as reported in other parts of the American continent. Meanwhile, this trial supports the finding that supplementation in general, and 1.5% BW in particular, is a suitable strategy to economically sustain animal performance and reduce the dependence on AH treatments, as suggested by Torres-Acosta et al. [35].

## 5. Conclusion

An improvement in kid resilience and resistance against GIN was obtained when maize supplementation was provided as a proportion of kid BW, as compared with a fixed amount of supplement (108 g/d). Best results in terms of production and resistance against natural GIN infection were obtained with the highest supplementation level evaluated (1.5% BW), which was economically viable. These results underline the importance of adjusting both the nature and quantity of supplementation according to the features of the forage available in the grazing area and expected host nutritional requirements.

## 6. Conflict of interest

The authors have no financial or personal relationships with other people or organizations that could inappropriately influence or bias the content of this paper.

## References

[1] AFRC (Agricultural and Food Research Council). 1993 Energy and protein requirements of ruminants. CAB International: Wallingford, UK.

[2] Balic A, Bowles VM, Meeusen ENT. 2000 The immunobiology of gastrointestinal nematode infections in ruminants. Advances in Parasitology, 45, 181–241. 10751941 10.1016/s0065-308x(00)45005-0

[3] Barger IA, Le Jambre LF, Georgi JR, Davies HI. 1985 Regulation of *Haemonchus contortus* populations in sheep exposed to continuous infection. International Journal for Parasitology, 15, 529–533. 4066146 10.1016/0020-7519(85)90049-9

[4] Barger IA. 1987 Population regulation in trichostrongylids of ruminants. International Journal for Parasitology, 17, 531–540. 3294665 10.1016/0020-7519(87)90129-9

[5] Barth D, Visser MX. 1991 Magen-Darm-nematoden des rindes. Diagnostischer atlas 284 Einzelabbildungen. Ferdinand Enke Verlag: Stuttgart, Germany.

[6] Benjamin MM. 1991 Manual de patología clínica en veterinaria (Handbook of clinical pathology in veterinary medicine). Limusa: México, DF.

[7] Blackburn HD, Rocha JL, Figueiredo EP, Berne ME, Vieira LS, Cavalcante AR, Rosa JS. 1991 Interaction of parasitism and nutrition and their effects on production and clinical parameters in goats. Veterinary Parasitology, 40, 99–112. 1763494 10.1016/0304-4017(91)90086-b

[8] Blackburn HD, Rocha JL, Figueiredo EP, Berne ME, Vieira LS, Cavalcante AR, Rosa JS. 1992 Interaction of parasitism and nutrition in goats: effects on haematological parameters, correlations, and other statistical associations. Veterinary Parasitology, 44, 183–197. 1466129 10.1016/0304-4017(92)90116-q

[9] Bowman DD, Lynn RC. 1999 Georgis’ Parasitology for Veterinarians, 7th edn WB Saunders: Philadelphia, Pennsylvania.

[10] Coop RL, Kyriazakis I. 1999 Nutrition-parasite interaction. Veterinary Parasitology, 84, 187–204. 10456415 10.1016/s0304-4017(99)00070-9

[11] Coop RL, Kyriazakis I. 2001 Influence of host nutrition on the development and consequences of nematode parasitism in ruminants. Trends in Parasitology, 17, 325–330. 11423375 10.1016/s1471-4922(01)01900-6

[12] Corticelli B, Lai M. 1963 Studies on the technique of culture of infective larvae of gastrointestinal strongyles of cattle. Acta de Medicina Veterinaria Napoli, 9, 347–357.

[13] Datta FU, Nolan JV, Rowe JB, Gray GD, Crook BJ. 1999 Long-term effects of short-term provision of protein-enriched diets on resistance to nematode infection, and live-weight gain and wool growth in sheep. International Journal for Parasitology, 29, 479–488. 10333332 10.1016/s0020-7519(98)00209-4

[14] Estrada-Liévano JM, Sandoval-Castro CA, Ramírez-Avilés L, Capetillo-Leal CM. 2009 *In vitro* fermentation efficiency of mixtures of *Cynodon nlemfuensi*s, *Leucaena leucocephala* and two energy sources (maize or sugar cane molasses). Tropical and Subtropical Agroecosystems, 10, 497–503.

[15] Flores JS, Espejel I. 1994 Tipos de vegetación de la península de Yucatán (Vegetation types of the Yucatan Peninsula). Etnoflora yucatense. Fascículo III. Universidad Autónoma de Yucatán: Mérida, Yucatán, México.

[16] Galicia-Aguilar HH, Rodríguez-González LA, Capetillo-Leal CM, Cámara-Sarmiento R, Aguilar-Caballero AJ, Sandoval-Castro CA, Torres-Acosta JFJ. 2012 Effects of *Havardia albicans* supplementation on feed consumption and dry matter digestibility of sheep and the biology of *Haemonchus contortus*. Animal Feed Science and Technology, 176, 178–184.

[17] González-Pech PG, Torres-Acosta JFJ, Sandoval Castro CA. 2014 Adapting a bite-coding grid for small ruminants browsing a deciduous tropical forest. Tropical and Subtropical Agroecosystems, 17, 63–70.

[18] Gutiérrez SI, Torres-Acosta JFJ, Aguilar-Caballero AJ, Cob-Galera L, May-Martínez M, Sandoval-Castro CA. 2003 Supplementation can improve resilience and resistance of browsing criollo kids against nematode infections during the wet season. Tropical and Subtropical Agroecosystems, 3, 537–540.

[19] Huirne RBM, Dijkhuizen AA. 1997 Basic methods of economic analysis, in Animal health economics principles and applications. Dijkhuizen AA, Morris RS, Editors. Post Graduated Foundation in Veterinary Science, University of Sydney: Australia p. 25–40.

[20] Hristov NA, Ropp KJ. 2003 Effect of dietary carbohydrate composition and availability on utilization of ruminal ammonia nitrogen for milk protein synthesis in dairy cows. Journal of Dairy Science, 86, 2416–2427. 12906060 10.3168/jds.S0022-0302(03)73836-3

[21] Hoste H, Torres-Acosta JFJ, Paolini V, Aguilar-Caballero A, Etter E, Lefrileux Y, Chartier C, Broqua C. 2005 Interactions between nutrition and gastrointestinal infections with parasitic nematodes in goats. Small Ruminant Research, 60, 141–151.

[22] Hoste H, Martínez-Ortiz-de-Montellano C, Manolaraki F, Brunet S, Ojeda-Robertos N, Fourquaux I, Torres-Acosta JFJ, Sandoval-Castro CA. 2012 Direct and indirect effects of bioactive tannin rich tropical and temperate legumes against nematode infections. Veterinary Parasitology, 186, 18–27. 22188981 10.1016/j.vetpar.2011.11.042

[23] Knox MR, Torres-Acosta JFJ, Aguilar-Caballero AJ. 2006 Exploiting the effect of dietary supplementation of small ruminants on resilience and resistance against gastrointestinal nematodes. Veterinary Parasitology, 139, 385–393. 16765520 10.1016/j.vetpar.2006.04.026

[24] Landa-Cansigno E, Torres-Acosta JFJ, Aguilar-Caballero AJ, Vargas-Magaña JJ, Schneck M, Friesendalh E, Cob-Galera L. 2005 Effect of maize or sugarcane molasses on resilience and resistance of criollo kids against gastrointestinal nematodes, in Proceedings of the XX Reunion Nacional sobre Caprinocultura 5th–7th October 2005 Culiacán, Sinaloa, México Asociación Mexicana de Producción Caprina A.C p. 495–499.

[25] Martínez-Ortiz-de-Montellano C, Vargas-Magaña JJ, Aguilar-Caballero AJ, Sandoval-Castro CA, Cob-Galera L, May-Martínez M, Miranda-Soberanis R, Hoste H, Cámara-Sarmiento R, Torres-Acosta JFJ. 2007 Combining the effects of supplementary feeding and copper oxide needles for the control of gastrointestinal nematodes in browsing goats. Veterinary Parasitology, 146, 66–76. 17400391 10.1016/j.vetpar.2007.02.012

[26] Martínez-Ortíz-de-Montellano C, Vargas-Magaña JJ, Canul-Ku HL, Miranda-Soberanis R, Capetillo-Leal C, Sandoval-Castro CA, Hoste H, Torres-Acosta JFJ. 2010 Effect of a tropical tannin-rich plant *Lysiloma latisiliquum* on adult populations of *Haemonchus contortus* in sheep. Veterinary Parasitology, 172, 283–290. 20605336 10.1016/j.vetpar.2010.04.040

[27] MAFF (Ministry of Agriculture Fisheries and Food). 1986 Helminthology, in Manual of veterinary parasitological laboratory techniques. Her Majesty’s Stationary Office London, UK p. 1–67.

[28] Petrie A, Watson P. 2006 Statistics for veterinary and animal science, 2nd edn Blackwell Publishing: Oxford, UK p. 158–190.

[29] Poppi DP, Sykes AR, Dynes RA. 1990 The effect of endoparasitism on host nutrition – the implications for nutrient manipulation. Proceedings of the New Zealand Society of Animal Production, 50, 237–243.

[30] Retama-Flores C, Torres-Acosta JFJ, Sandoval-Castro CA, Aguilar-Caballero AJ, Cámara-Sarmiento R, Canul-Ku HL. 2012 Maize supplementation of pelibuey sheep in a silvopastoral system: fodder selection, nutrient intake and resilience against gastrointestinal nematodes. Animal, 6, 145–153. 22436162 10.1017/S1751731111001339

[31] Ríos G, Riley JA. 1985 Preliminary studies on the utilization of the natural vegetation in the henequen zone of Yucatan for the production of goats. I. Selection and nutritive value of native plants. Tropical Animal Production, 10, 1–10.

[32] Torres-Acosta JFJ, Hoste H. 2008 Alternative or improved methods to limit gastro-intestinal parasitism in grazing sheep and goats. Small Ruminant Research, 77, 159–173.

[33] Torres-Acosta JFJ, Jacobs DE, Aguilar-Caballero AJ, Sandoval-Castro CA, May-Martínez M, Cob-Galera LA. 2004 The effect of supplementary feeding on the resilience and resistance of browsing Criollo kids against natural gastrointestinal nematode infections during the rainy season in tropical Mexico. Veterinary Parasitology, 124, 217–238. 15381302 10.1016/j.vetpar.2004.07.009

[34] Torres-Acosta JFJ, Jacobs DE, Aguilar-Caballero AJ, Sandoval-Castro C, Cob-Galera L, May-Martínez M. 2006 Improving resilience against natural gastrointestinal nematode infections in browsing kids during the dry season in tropical Mexico. Veterinary Parasitology, 135, 163–173. 16203095 10.1016/j.vetpar.2005.08.009

[35] Torres-Acosta JFJ, Sandoval-Castro CA, Hoste H, Aguilar-Caballero AJ, Cámara-Sarmiento R, Alonso-Díaz MA. 2012 Nutritional manipulation of sheep and goats for the control of gastrointestinal nematodes under hot humid and subhumid tropical conditions. Small Ruminant Research, 103, 28–40.

